# Towards a Standard Mixed-Signal Parallel Processing Architecture for Miniature and Microrobotics

**DOI:** 10.6028/jres.119.020

**Published:** 2014-09-08

**Authors:** Brian M Sadler, Sebastian Hoyos

**Affiliations:** Army Research Laboratory, Adelphi, MD 20783; Texas A&M University, Department of Electrical and Computer Engineering, College Station, TX 77843

**Keywords:** analog-to-digital conversion, communications, control, mixed-signal architecture, mixed-signal processing, perception, robotics, sensing, signal processing

## Abstract

The conventional analog-to-digital conversion (ADC) and digital signal processing (DSP) architecture has led to major advances in miniature and micro-systems technology over the past several decades. The outlook for these systems is significantly enhanced by advances in sensing, signal processing, communications and control, and the combination of these technologies enables autonomous robotics on the miniature to micro scales. In this article we look at trends in the combination of analog and digital (mixed-signal) processing, and consider a generalized sampling architecture. Employing a parallel analog basis expansion of the input signal, this scalable approach is adaptable and reconfigurable, and is suitable for a large variety of current and future applications in networking, perception, cognition, and control.

## 1. Introduction

Over the last decade there has been an increasing convergence of communications, processing and computation, control, and mobility into miniaturized devices and platforms. This brings together many signal processing (SP) tasks across sensing, perception and navigation, control systems, and networking. Significant progress in SP has occurred for all of these disciplines individually, and some combined tools and analyses are also now emerging. The SP toolchest is filling with a variety of algorithms, giving the system designer an increasingly sophisticated palette to paint from.

While this is certainly good news, the mini- or micro-system designer faces a confusingly large array of tradeoffs and possibilities. For example, some of these are well understood in the context of wireless sensor networks (WSNs), albeit typically with limited mobility and single-minded application [1, 2]. The fundamental currencies of trade are energy (Joules), lifetime (seconds), and technology cost (dollars per unit, or bill of materials). Technology miniaturization and integration are expensive, and effective R&D investment requires an understanding of theoretical and technological tradeoffs, and fundamental limits. A system design will attempt to balance the Joules-second-cost trades in a way that results in an affordable and reasonably satisfying implementation. The study of animals, insects, and bacteria yields living proof of principle and provides significant insight into mini and microrobotic architecture [3–5], spanning inches to micro-scales of a mm or less [6].

The implementation question is tightly coupled with current and future trends in circuit fabrication. The high cost of the iterative circuit design and fabrication cycle seriously hinders major advances in micro and miniature robotic systems. While there has been some significant advancement in producing one-off mechanical and structural components at relatively low cost (e.g., 3-D printing), this is generally not true for electronics due to the very high cost of circuit design. Instead, some specialized components are developed, while a system must rely on available commercial devices (especially those in mass production such as for entertainment and communications).

These trends motivate the study of signal processing architectures that have some general utility for future systems such as miniature robotics. In this paper we consider the combination of analog and digital, or mixed-signal, processing as a general reconfigurable interface with an analog input and digital information output. A parallel sampling architecture is described that is both fundamental and reconfigurable, and we briefly consider some signal processing applications with this as a mixed-signal front end. This architecture is a good candidate as a standardized approach for a broad array of applications in future robotic systems, and can be viewed as a logical parallel extension of a conventional sampler. As such, it is especially motivated when the conventional approach is limiting, including high bandwidth, multi-channel, and reconfigurable cases.

This paper published in the *Journal of Research of NIST* was contributed by a speaker at the 2008 NIST Workshop on MEMS Robotics. At the workshop, speakers described unique MEMS robots; applications of MEMS robots to micro-assembly and medicine; and descriptions of requirements for communicating with MEMS robots. This paper addresses signal processing in the mixed analog/digital, low-power environment of a MEMS robot which places severe limits on power and logic. This paper was edited by Richard A. Allen and Craig D. McGray, both of NIST, who co-chaired the 2008 Workshop.

## 2. Reconfigurable Mixed-Signal Sampling

### 2.1 Analog and Digital

**Fig. 1:**
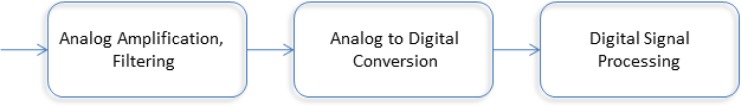
Conventional mixed-signal analog-to-digital sampling architecture.

On the one extreme, a conventional sampling architecture employs an analog-to-digital converter (ADC) driven in series by a band-limited analog source, and produces samples at or above the Nyquist rate (see Fig. 1). The analog interface typically consists of an automatic gain control and an anti-aliasing filter preceeding the ADC, and often includes mixing for frequency translation. As a stand alone sub-system, the goal is to faithfully represent the analog signal in the digital domain. This architecture has prospered for several decades, and its limitations are well known [7, 8]. Fundamental is a tradeoff between dynamic range (i.e., the effective number of ADC bits) versus bandwidth for a fixed power consumption target. The architecture is not very flexible with regard to reconfiguring the sampling rate (signal bandwidth). Other issues include linearity and power consumption, which become increasingly prohibitive when the sampling rates are pushed to GHz.

A commonly employed figure of merit (FOM) for ADC technology is the power in pico-Joules per conversion, given by [8]

$$\mathrm{FOM}=\frac{P}{2^{\mathrm{ENOB}}(2B)},\;(\mathrm{pJ}/\text{conversion})$$ (1)

where *P* is the device power, ENOB = effective number of bits, and *B* is the signal bandwidth (henc 2*B* is the Nyquist sampling rate). State of the art numbers for *FOM* are roughly 1 pJ per conversion, with many commercially available devices in the 1 to 20 range.^1^ This means that as samplers move to GHz, the required device power moves towards many Watts.

The all-digital view of SP provides generality and programmability (although typical digital signal processing (DSP) texts ignore the issues cited above, such as nonlinearities and timing jitter), and DSP has obviously yielded dramatic advancement in the application of computing in small devices. We all envision further advances in robotics and related areas that rely on sophisticated SP, e.g., software-defined radio is viewed by many as ultimately providing an adaptive cognitive engine for the radio SP. However, it is often not noted that device programmability is relative to the technology employed. DSP circuits are roughly limited to a few technology bins (GPP, FPGA, DSP, ASIC^2^). These can be thought of as trading power for programmability. More specialized circuits (DSP, ASIC) perform dedicated tasks with lower power and/or higher bandwidth. But, these specialized circuits incur significant design cost, and so their availability is limited and generally driven by mass market production.

On the other extreme is dedicated analog processing, such as a correlator or spectrum analyzer. Here the goal is to extract some information, rather than to preserve the input signal, with the analog providing a computational engine for some detection, estimation, or classification task. The output will then typically be digitized, but at a rate commensurate with the estimation update, which may be orders of magnitude less than the corresponding Nyquist sampling rate matched to the original signal bandwidth. Analog systems have traditionally traded dynamic range for bandwidth, and generally lack reconfigurability (which as we noted is true for ADCs). Over the preceeding few decades, as technology scaling has provided ADCs and digital circuits with higher speed and lower power consumption, the conventional architecture in Fig. 1 has replaced its analog counterpart; e.g., in radio and communications, radar, imaging, and video.

Of course, analog processing is certainly not gone. Interesting examples include phase-lock loops (PLLs), code synchronization [9], and computing on graphs with probabilities that includes a very broad class of algorithms [10]. Computing on graphs with probabilities includes the complex iterative calculations for decoding of turbo codes in a dedicated analog circuit [11, 12], which may provide significant energy savings; such a circuit is broadly applicable in wireless communications. We should also not forget that simple traditional analog circuits can be extremely power efficient and sufficiently accurate, e.g., an FM radio demodulator. Overall, the trends in areas such as wireless sensor networks and mini/micro-robotics have created a renewed interest in very low power analog signal processing, and this trend is accelerating as systems designers focus more and more on the greening of technology.

### 2.2 Mixed-Signal Sampling

To move beyond the conventional serial architecture we consider a generalized sampling scheme. The goal is to define a multi-purpose mixed signal architecture that is broadly applicable and of high enough utility to justify circuit design costs. What are the desirable features of such a generalized sampler? The approach should handle high bandwidths, but at the same time be scalable, i.e., allow for bandwidth reconfiguration. It should as much as possible avoid the dynamic range versus bandwidth limiting tradeoffs inherent in the serial ADC architecture. Bearing in mind that higher rates generally require more power, it should seek to provide an adaptable compromise between power consumption and high rate operation. It should provide some amount of parallelization, e.g., be applicable to multi-dimensional problems such as antenna array processing or imaging. It should provide parallel digital output streams to facilitate parallel DSP, and the parallelization should be appropriate to a large variety of applications and DSP algorithms (communications, sensing, control). The architecture should couple with and incorporate analog processing as desired, i.e., it should be amenable to mixed-signal processing as well as sampling. This provides a degree of robustness, e.g., enabling analog filtering for removal of interferers. Finally, it should be as robust as possible to technology imperfections.

**Fig. 2:**
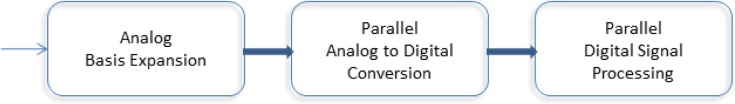
Generalized sampling via basis expansion.

As one possible architecture, consider the generalized sampling scheme in Fig. 2. Fundamentally, we first decompose the signal via analog processing, and then sample the decomposition in parallel. Many decompositions and basis expansions are possible; in the following we highlight basis function families that are amenable to lower complexity circuit implementation, as well as possible future extensions.

**Fig. 3:**
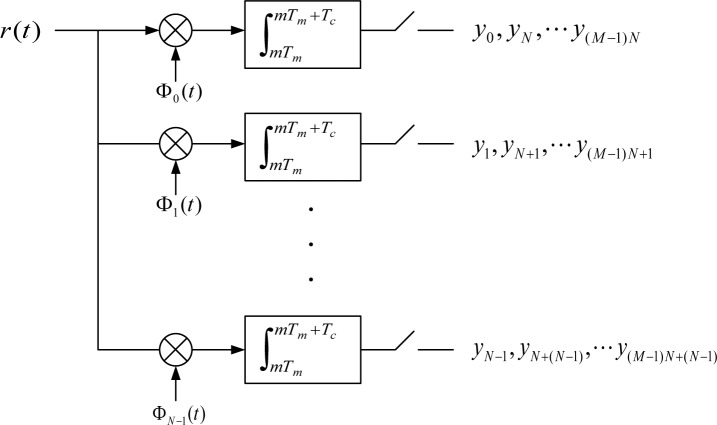
Mixed-signal basis expansion architecture.

A particular realization of this scheme is shown in Fig. 3 [13]. The input analog signal *r*(*t*) proceeds via *N* parallel paths. We consider the real-valued signal case for simplicity; the generalization to complex-valued is straightforward, with a doubling of the number of parallel paths. The output of the *n*th path, *n* = 0,1, …, *N* − 1, is given by

$$y_{mN+n}=\int_{mT_{m}}^{mT_{m}+T_{c}}r(t)\Phi_{n}(t)dt,$$ (2)

where Φ*_n_* (*t*) is the *n*th basis function, and *T_c_* is the integration interval length. Here, integer counter *m* = 0,1,… advances with each integration time interval of *T_c_* seconds. Thus, the *N* parallel integrators yield a new vector of analog samples (that are continuously variable in amplitude) every *T_c_* s, indexed by *m*, and given by

$$y_{m}={\left[y_{mN},y_{mN+1},\cdots ,y_{mN+(N-1)}\right]}^{T},\;m=0,1,\cdots .$$ (3)

The figure shows *M* basis expansion intervals, 0 ≤ *m* ≤ *M* −1, corresonding to *MT_c_* seconds elapsed time, although there is no limit on *M*

The underlying assumption is that the input signal can be expanded in the chosen basis as

$$r(t)=\sum_{i=0}^{N-1}a_{i}\Phi_{i}(t).$$ (4)

Discussion of the choice of the basis functions is deferred until Sec. 3. Equation (4) is generally an approximation, whose truncation error can be made arbitrarily small. Suppose the input signal is ideally bandlimited to bandwidth *B*. Then, the fundamental sampling relationship is

$$N\geq \left\lceil T_{c}B\right\rceil$$ (5)

where is ⌈⋅⌉ the ceiling function. That is, the number of parallel sampling paths must be greater than or equal to the integration time-bandwidth product *T_c_B*. This ensures complete information capture. In practice, if the input is essentially bandlimited through a pre-conditioning filter, then (4) holds to high accuracy when (5) is satisfied. More generally in a non-bandlimited scenario, *P* signal parameters can be obtained using linear estimation (e.g., using least squares to minimize an appropriate mean square error criterion) if at least *P* samples are produced with the parallel scheme. Oversampling and engineering margin can be incorporated by suitable variation of *N*, *T_c_ B.*

Equation (5) reveals the reconfigurability of this approach. For example, with the number of parallel hardware paths *N* fixed, changes in the input signal bandwidth *B* can be accomodated by changing *T_c_*. This leads to practical tradeoffs between hardware complexity and device bandwidth [13].

The analog voltages in *y_m_* may be fed to *N* parallel ADCs, each of which are now running at a relaxed rate as compared to the full bandwidth Nyquist rate. Thus, the architecture provides flexibility between bandwidth and digital dynamic range by first parallelizing the output. Note also that, rather than feeding *y_m_* to ADCs, we have the option of further processing in the analog domain, e.g., via a switched-capacitor approach [14].

As shown in Fig. 3, the processing intervals have length *T_c_* s and do not overlap, which leads to an implied rectangular window weighting in the time domain over each interval. In practice, it is beneficial to instead allow some small overlap between processing intervals, and to tune the window weighting as desired, to avoid sharp switching transient effects at the interval boundaries, and to obtain some desired frequency roll-off response. One such scheme employs a trapezoidal window weighting, i.e., with linear slope at the interval boundaries. A circuit implementation is described in [15, 16]. By preserving symmetry in the window rise and fall shaping, orthogonality can be preserved from segment to segment, ensuring that each successive basis expansion is independent [17].

Fundamental to any sampling scheme is quantization error. Here, the quantization occurs in the basis coefficient sampling, rather than in the time domain, and this relaxes somewhat the sensitivity to quantization. And, the parallelization allows for increased quantization levels as desired, e.g., knowledge of the signal can be employed to optimally allocate bits via vector quantization [13]. Other error sources arise due to device imperfections, mismatch between signal paths, timing jitter, and so on. The basis expansion architecture has relatively good robustness to such error sources.

It is interesting to compare this approach with two more traditional ADC architectures, using either time-interleaving of ADCs, or parallel analog bandpass filters continuously feeding ADCs. The time-interleaving structure [18–22] and the multi-channel filter-bank approach [23, 24] have received the most attention, although at high sampling rates the power consumption of these topologies is still high relative to desired applications in the mini and micro worlds. The time-interleaved approach suffers from the need for a full-bandwidth sample and hold circuit for the interleaved ADCs, while the parallel filter approach leads to significant issues with filter design and calibration. The parallel basis expansion approach is similar, but also fundamentally different than, a parallel filter bank [13].

In addition to time-interleaving, a frequency-interleaved architecture is also possible. Time-interleaved versus frequency-interleaved architectures for wideband parallel mixed signal sampling and processing are contrasted in [25], in the context of sensing for cognitive radios. See also [26] for a frequency domain implementation, based on an analog switched capacitor FFT computation that is extremely energy efficient when compared with a conventional full bandwidth sampler followed by an FFT algorithm in DSP. This can be regarded as a basis expansion architecture, using analog to carry out an FFT, followed by parallel sampling of the complex-valued FFT coefficients [26].

Calibration is generally needed in parallel architectures due to variations in manufacturing, slight offsets between channels, nonlinearities, and so on. This can be accomplished via an open or closed loop approach, e.g., using simple LMS type DSP algorithms [27]. Note that the calibration can also be built into an application, and calibration requirements relaxed, when not trying to obtain high resolution samples but instead carrying out some detection or estimation task such as those described in the next section.

## 3. Mixed-Signal Application

Basis expansion is ubiquitous in signal processing, and a very large variety of problems and algorithms are compatible with basis decomposition as a first step. We have only to consider the short-time Fourier transform as a basis expansion to realize this is true. Let us assume an orthonormal basis with *N* basis functions, although there is no restriction to orthogonality. Then,

$$\int_{0}^{T_{c}}\Phi_{i}(t)\Phi_{j}(t)dt=\{\begin{array}{c} 1,i=j\\ 0,i\neq j \end{array},\;0\leq i,j\leq N-1.$$ (6)

From a circuit implementation perspective, two appealing choices for the basis functions Φ*_i_* (*t*) are (i) those that consist of binary waveforms, and (ii) complex exponentials. Tones and binary signals are straightforward to produce in dedicated simple circuits,^3^ with relatively low power consumption, avoiding the use of general purpose digital to analog conversion (DAC) to produce the Φ*_i_* (*t*) waveforms. Employing complex exponentials results in the short-time Fourier transform with *N* coefficients, and there are many options for binary bases. More general non-binary basis functions can be employed, presumably at the cost of more complex and higher power circuitry, so there is a tradeoff in circuit complexity versus generality in choice of Φ*_i_* (*t*).

As a fundamental processing example using this architecture, consider matched filtering (template matching, correlation). A matched filter response is easily calculated following the basis expansion. Assuming (6), then the scalar matched filter output is given by

$$a=\sum_{m=0}^{M-1}\sum_{n=0}^{N-1}R_{m}(n)G_{m}^{*}(n)$$ (7)

where *R_m_* (*n*) are the basis coefficients from the received signal *r*(*t*), and $G_{m}^{*}(n)$ are the basis coefficients of the locally generated matched filter template. The summation is over the basis index 0 ≤ *n* ≤ *N* −1, and the time index 0 ≤ *m* ≤ *M* −1, corresponding to signal duration of *MT_c_* s.

The matched filter easily generalizes to communications receivers, incorporating channel estimation and equalization. The reconfigurability with regard to signal bandwidth *B* enables a multi-standard radio receiver front end [15, 16]. Linear receivers, such as minimum mean-square-error (MMSE) and zero-forcing, are easily incorporated as enhanced solutions with higher complexity than the truncated matched filter solution in (7). These can be used in a variety of wideband and ultra-wideband receivers [28–30]. The use of complex exponential basis functions is well suited to multi-carrier (OFDM) receivers, and we note that the number of basis elements *N* may be as small as *N* = 2 and is *not* required to be equal to the number of carriers (which may be in the hundreds) [31]; it is only required that condition (5) holds with respect to the entire OFDM signal bandwidth *B*. The architecture also naturally lends itself to cognitive radio, for example, employing wideband spectrum sensing and signal analysis to support smart networking and dynamic spectrum access techniques [32, 26, 25].

The basis expansion approach can be adapted to compressive sensing by randomizing the basis functions. For example, sparsity is often inherent such as in the frequency domain in wireless communications [33] and the wavelet-domain for images [34]. A compressive sensing analog front-end utilizes basis functions Φ*_i_* (*t*) that are pseudo-random and emulate white noise [35, 36]. By mixing the input signal with a randomized basis function, the signal is randomized and the information in each channel spreads over the entire bandwidth. Depending on the sparsity level, the number of basis functions can be dramatically reduced, yielding an effective sampling rate that is significantly below the Nyquist rate for the full bandwidth [37]. Given the samples from the compressive sensing channels, the input signal can be reconstructed using a variety of regularized optimization algorithms [38–40]. It is also possible to estimate second-order statistics from the compressive samples directly, avoiding signal reconstruction, e.g., see [41]. However, care must be taken with regard to loss of signal energy, which is generally proportional to the reduction in sampling rate with respect to the Nyquist rate, so that the SNR can be significantly reduced. The SNR reduction is more of an issue in radio and radar problems, and less of an issue in imaging, for example.

Analog filtering can also be incorporated into the basis decomposition to remove interference. For example, knowledge of the signal and/or interference subspace can be used to tune the basis and optimally reject undesired components while preserving the signal [42]. However, this may deviate from the simpler binary or exponential basis functions.

**Fig. 4:**
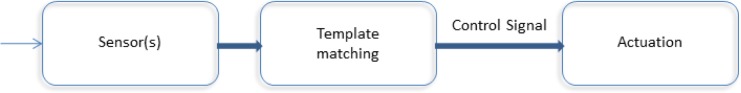
Complex control for mobility can be achieved by parallel template matching to sensor outputs. The control response is a function of the template matching weights. This architecture commonly occurs in nature.

Template matching and basis decomposition are also central to sensing and control problems, e.g., see Fig. 4. This architecture is related to, and inspired by, neural and biological cognitive and control functions. Neural processing leads to matched filtering applied to sensory outputs that may have already undergone some preparatory processing [43]. For example, consider visuomotor convergence, i.e., rapid processing and convergence of visual sensory information with flight control as occurs in insects and animals [44]. Based on image flow computation, image flow kernels (i.e., templates) are compared (i.e., matched filtered) and the matched filter outputs produce feedback terms for flight control. More generally, the link with template matching/correlation and neural processing is a very strong one [45], and this will continue to lead to advances in processing architectures for miniature devices.

The last few decades have seen significant progress in both minimally invasive surgery and medical sensing systems such as magnetic resonance imaging (MRI), although these two areas have not merged into real-time clinical application. As medical array-based sensing systems move towards massive numbers of channels to drastically reduce scan times [46–49] there is a correspondingly significant increase in hardware complexity (electrodes, RF coils, RF receivers, and so on), and the aggregated data rates become very high. This results in prohibitively high power consumption and places limits on the number of channels. In addition to sensors that are entirely external to the patient, there is a strong motivation to develop a combination of internal and external sensors, such as might be employed during surgical procedures. Robotic capsules that can be ingested are being actively investigated, especially for endoscopic and gastrointestinal track diagnosis, with potential to noninvasively deliver surgical tools and therapy [50–53]. This may lead to actively controlled motion, and self-assembling robotic elements [54]. These devices will require miniaturized processing, control, and communications along the lines we have described above.

Another medical example is the integration of many neural spike electrode array sensors in a chip, which comes with power consumption and heat dissipation issues [55–59]. Tetherless implantation is highly desirable, incorporating high bandwidth wireless communications, and ultimately including signal processing on the device. These large sensor arrays may require very agressive sub-picosecond clock-jitter specification, and will be sensitive to channel response mismatch and nonlinearities. Such an array may include 1000 electrodes or more, each requiring analog-to-digital conversion from a separate parallel channel. Consequently, new approaches are needed for efficient channel multiplexing that go beyond traditional time-interleaved digital conversion.

One emerging alternative approach is asynchronous sampling. This contrasts with the architectures discussed above, in that samples are not taken periodically. This can be viewed as a form of compressive sampling. For example, asynchronous sampling has been proposed in the context of low-power ultrasound imaging, by incorporating a compressed sensing framework that reduces hardware complexity and power consumption of the entire multichannel beamforming and signal processing chain [60, 61]. This relies on a continuous-time ternary encoding scheme that converts pulsed signal variations to high-rate ternary timing signals. Thus the asynchronous approach is particularly relevant to pulsed signals, i.e., signals that are sparse in time.

## 4. Conclusion

The outlook for miniature and microrobotic systems is significantly enhanced by advances in sensing, signal processing, and control. Looking towards future implementations, we have considered a generalized alternative to the conventional sampling and DSP approach. We are likely to see a variety of implementations spanning analog to full DSP, with most encompassing both to some degree. With this in mind, it is good to keep sight of fundamental SP goals that span virtually all applications, including signal decomposition and parallelization, correlation and matched filtering, spectrum estimation, and control based on applying these functions to sensory outputs. In the longer term, SP architectures are very likely to employ more and more (even “massively”) parallel processing based on cognitive neural-inspired ideas, and incorporating a variety of sensors and actuators. The generalized mixed-signal basis decomposition and sampling architecture described here may provide a good standard interface and reconfigurable approach suitable to a wide variety of such applications.

## Footnotes

1 Calculation of the FOM is complicated by an accurate measure of the ENOB, as well as associated processing such as the need for digital calibration circuits or signal pre-conditioning that add to power consumption *P*.

2 General purpose processor, field programmable gate array, digital signal processor, application specific integrated circuit.

3 There are difficulties associated with generating multiple tones that are locked with the desired frequency and phase offsets.
